# Pediatric Cutaneous Anaplastic Lymphoma Kinase-Positive Histiocytosis with *DCTN1::ALK* Fusion: A Case Report and Literature Search

**DOI:** 10.3390/diagnostics15091057

**Published:** 2025-04-22

**Authors:** Kristóf Levente Korpás, Attila Mokánszki, Lívia Beke, Gábor Méhes, Yi-Che Chang Chien

**Affiliations:** Department of Pathology, University of Debrecen Clinical Centre, 4032 Debrecen, Hungary

**Keywords:** histiocytosis, *DCTN1*, *ALK*, gene fusion

## Abstract

**Background and Clinical Significance**: Anaplastic lymphoma kinase (ALK)-positive histiocytosis is a relatively novel entity, affecting single or multiple organ systems; it is characterized by aggregates of neoplastic cells of the histiocytic lineage, harboring molecular alterations in the *ALK* gene and exhibiting excellent response to systemic tyrosine kinase inhibitors. **Case presentation**: Herein, we present a pediatric case with cutaneous-only involvement: the 6-month-old male patient presented with an elevated, tan-colored lesion on his left forearm. Following surgical excision, histopathological evaluation reported spindle cells with wide eosinophilic cytoplasm and Touton-type giant cells. The tumor cells were positive for CD163, ALK, phosphorylated ERK, and cyclin D1. Fluorescent in situ hybridization revealed *ALK* rearrangement, whereas, upon next-generation sequencing, a *DCTN1::ALK* fusion was identified. **Conclusion**: Our case serves as a great addition to the limited number of cases reported in the literature, and it represents the first published pediatric case with the rare *DCTN1::ALK* fusion. The novelty of this genetic alteration and the lack of knowledge about its potential effects on the clinical aspects of ALK-positive histiocytosis highlight the importance of ancillary molecular testing, when available.

## 1. Introduction

The umbrella term ‘histiocytosis’ refers to diseases in which aggregates of dendritic cell-, monocyte-, or macrophage-derived cells are present throughout one or more organ systems, including the bone, skin, lung, lymphatic, hematopoietic, and nervous systems. It is known today that many histiocytoses share specific genetic aberrations, predominantly affecting the mitogen-activated protein kinase/extracellular signal-regulated kinase (MAPK-ERK) signaling pathway; however, activating mutations in the phosphatidylinositol-3-kinase/Akt kinase/mammalian target of rapamycin (PI3K/AKT/mTOR) signaling pathway or receptor tyrosine kinases, e.g., colony-stimulating factor 1 receptor (CSF1R) or neurotrophic receptor tyrosine kinase 1 (NTRK1) have also been described [1].

Anaplastic lymphoma kinase (ALK) is a receptor tyrosine kinase physiologically found mainly in neuronal tissue [2]. It activates various downstream signaling pathways, including the MAPK-ERK, PI3K-Akt, or Janus kinase/signal transducer and activator of transcription (JAK-STAT) signaling pathways, leading to the transcription of such effector genes as cyclin D1 [3]. When a certain fraction of the *ALK* gene fuses with specific partner genes, it may become constitutively activated, even without activating ligands, thus turning oncogenic, leading to excessive and uncontrolled proliferation of the affected cells. The most common fusion partners are *NPM* and *TPM3* genes in anaplastic large cell lymphomas and *EML4*, *TFG*, and *KIF5B* genes in pulmonary adenocarcinomas; however, over 90 fusion partners have been detected to date [4,5,6,7,8]. ALK-positive histiocytosis was first described in 2008 by Chan et al., and 39 cases have been reported in the literature; most of them harbor *KIF5B-ALK* gene fusions [9,10]. The disease usually has an excellent response to systemic ALK inhibition.

Herein, we present the case of a 6-month-old infant with mono-systemic, cutaneous ALK-positive histiocytosis harboring *DCTN1::ALK* fusion.

## 2. Materials and Methods

Surgical specimens were received in a 4% phosphate-buffered formaldehyde solution (Sigma-Aldrich. St. Louis, MO, USA) and fixated overnight before macroscopic examination and grossing. Resection margins were inked with India ink. After formalin-fixed paraffin-embedded processing, 2 μm thick slides were made, followed by routine hematoxylin and eosin staining. Additional Gram and Grocott–Gomori stainings were also performed.

The immunohistochemical stainings are detailed in Table 1.

For the analysis of fluorescence in situ hybridization, the ZytoLight SPEC ALK/EML4 TriCheckTM Probe (ZytoVision GmbH, Bremerhaven, Germany) was utilized, according to the manufacturer’s protocol. The analysis was performed on 5 µm thick sections of FFPE samples. Deparaffinized sections (Q Path Safesolv, VWR, Debrecen, Hungary) were pretreated with pretreatment buffer, followed by proteolytic digestion using protease solution (MetaSystems, Altlussheim, Germany). Slide and probe codenaturation was carried out at 75 °C for 10 min, after which hybridization was conducted at 37 °C in a moist chamber for 16–18 h (StatSpin ThermoBrite, Abbott Molecular, Des Plaines, IL, USA). Post-hybridization washes were performed with 2X SSC/0.1% NP-40 for 5 min. The slides were then washed with 2X SSC/0.3% NP-40 at 74 °C for 3 min. After washing, the nuclei were counterstained with 4′,6-diamidino-2-phenylindole (DAPI, MetaSystems, Altlussheim, Germany). Scoring was performed using a Zeiss Axio Imager Z2 (Carl Zeiss, Cambridge, UK) fluorescence microscope, and the images were captured and analyzed by ISIS software (MetaSystems, Altlussheim, Germany).

For NGS library preparation, the Archer FusionPlex Core Solid Tumor Panel panel (Archer DX, Boulder, CO, USA) was used with the coverage of the following genes: *AKT1*, *ALK*, *AXL*, *BRAF*, *BRD3*, *BRD4*, *CTNNB1*, *CYSLTR2*, *DDR2*, *DNAJB1*, *EGFR*, *ERBB2*, *ERBB4*, *ERG*, *ESR1*, *FGFR1*, *FGFR2*, *FGFR3*, *GNA11*, *GNAQ*, *GNAS*, *H3F3A*, *HIST1H3B*, *HRAS*, *IDH1*, *IDH2*, *KEAP1*, *KIT*, *KRAS*, *LTK*, *MAP2K1*, *MAP3K3*, *MAP3K8*, *MET*, *MYB*, *MYBL1*, *NRAS*, *NRG1*, *NTRK1*, *NTRK2*, *NTRK3*, *NUTM1*, *PAX8*, *PDGFRA*, *PIK3CA*, *POLD1*, *POLE*, *PPARG*, *PRKCA*, *PRKCB*, *RAF1*, *RET*, *ROS1*, *STK11*, *TMPRSS2*, *TP53*, and *TRIM11*.

Anchored primers were applied for the known translocation partners, and reverse primers to hybridize with the sequencing adapters to identify breakpoints and partners. A total of 100–250 ng of RNA was loaded into the assay, after which first-strand cDNA synthesis was performed. Subsequently, a quantitative RT-PCR Pre-seq QC was carried out to define the yield of intact RNA in the samples. The final libraries were then quantified using the KAPA library quantification kit (Roche, Basel, Switzerland), diluted to a final concentration of 4 nM, and pooled by equal molarity.

For sequencing on the MiSeq System (MiSeq Reagent kit v3, 600 cycles), it was necessary to denature all the libraries by adding 0.2 nM NaOH and, subsequently, to dilute them to 40 pM with hybridization buffer from Illumina (San Diego, CA, USA). The final loading concentration was 10 pM libraries and 5% PhiX. The process of sequencing was conducted in strict accordance with the MiSeq instruction manual. Captured libraries were then subjected to a multiplexed sequencing process utilizing a paired-end run, thereby yielding 2 × 150 bp reads with a minimum depth of coverage of 250X. After this, trimmed fastq files were generated using MiSeq Reporter (Illumina, San Diego, CA, USA), and these files were then analyzed using Archer analysis software (version 7; Archer DX, Boulder, CO, USA). For the alignment, the human reference genome GRCh38 (equivalent UCSC version hg38) was built. Molecular barcode (MBC) adapters were used to count unique molecules and characterize sequencer noise, revealing mutations below standard NGS-based detection thresholds. The sequence quality for each sample was assessed, and the cutoff was set to 5% variant allele frequency (VAF). Translocations were identified when there were over five reads containing a fusion sequence, with these reads making up at least 10% of the total gene-specific primer reads. The gene fusion frequency was calculated for fusion transcript reads and the total reads ratio.

## 3. Case Report

### 3.1. Clinical Presentation, Management, and Follow-Up

**Figure 1:**
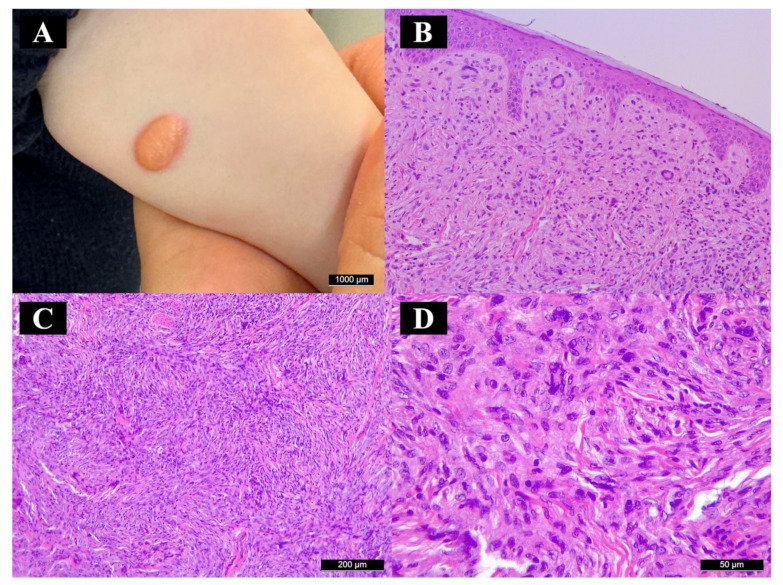
Macroscopic and histopathological features (hematoxylin–eosin). (**A**) The lesion appeared nodular and smooth-surfaced, with sharp edges and a tan color. (**B**–**D**) Microscopically, a dense dermal infiltrate of histiocytes and scattered Touton-type giant cells was seen.

The 6-month-old male infant presented with a 15 × 10 mm sized, elevated, orange-tan-colored, well-circumscribed, smooth-surfaced, firm nodule on his left forearm (Figure 1A). The lesion had appeared a few months prior and showed a rapidly growing tendency. He had no related symptoms. The lesion was excised in toto and submitted to histopathological evaluation. During the second postoperative week follow-up, the incision was healed, and no visible mass was visible or palpable. However, due to positive surgical margins, re-excision was performed on postoperative week 5, and negative surgical margins were achieved. The multidisciplinary oncology team recommended observation. Upon 6-month follow-up, no local or systemic recurrence was reported.

### 3.2. Histopathology and Immunohistochemistry

**Figure 2:**
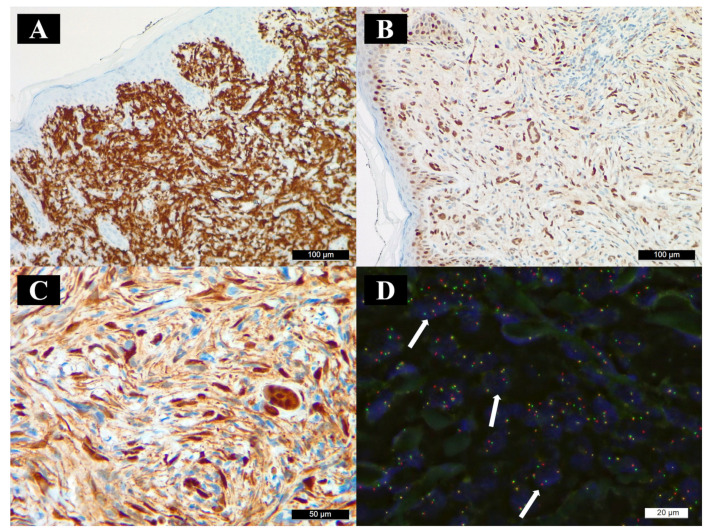
Immunohistochemistry and fluorescence in situ hybridization (FISH). (**A**) ALK, (**B**) cyclin D1, and (**C**) pERK immunoreactivity in the lesional cells. (**D**) FISH revealed ALK gene rearrangements (green—proximal to the ALK breakpoint region; red—distal to the *ALK* breakpoint region; white arrows point at representative nuclei with red and green signal separation, indicating *ALK* rearrangement).

On hematoxylin- and eosin-stained slides, a well-circumscribed nodule was observed, located primarily in the dermis, extending slightly into the subcutaneous fat. The nodule was composed of spindle cells with a wide eosinophilic cytoplasm and large, euchromatic nuclei. Touton-type giant cells and foci of lymphocytic infiltrates were seen dispersed in the lesion (Figure 1B–D). The lesional histiocytes were negative for S-100, HMB-45, CD10, and CD34, but positive for ALK and CD163. ALK immunoreactivity showed a diffuse granular intracytoplasmic staining pattern (Figure 2A). Fifty percent of the lesional cells showed nuclear positivity for cyclin D1 (Figure 2B); 80% showed nuclear or cytoplasmic positivity for phosphorylated ERK (pERK) (Figure 2C), and the MIB-1 labeling index was 40%. The overlying surface epithelium appeared unremarkable; however, the lateral and inferior surgical margins were positive. Gram and Grocott–Gömöri methenamine silver staining excluded microorganism presence in the lesion.

### 3.3. Molecular Findings

After detecting ALK immunopositivity, fluorescence in situ hybridization (FISH) was performed to assess possible *ALK* gene amplification and rearrangements (Figure 2D). In 52% of the cells, rearrangement of the *ALK* 2p23 region was detected.

Subsequently, next-generation sequencing (NGS) revealed *DCTN1::ALK* gene fusion, which is considered a gain-of-function alteration. The breakpoints included exon 26 in the *DCTN1* gene and exon 20 in the *ALK* gene. No mutations of *BRAF*, *EGFR*, *KIT*, *KRAS*, or *TP53* were detected (see Methods and Materials for a complete list of examined genes).

### 3.4. Imaging and Laboratory Findings

Post-operative computed tomography (CT) imaging of the thorax, magnetic resonance imaging (MRI) of the abdominal and pelvic region, and ^99m^Tc methylene-diphosphonate bone scintigraphy were obtained to assess possible systemic disease progression. MRI showed—as previously known—mild left-sided pyelectasia; however, all scans revealed no systemic disease manifestation.

The laboratory findings were unremarkable.

## 4. Discussion

The present study reports a pediatric case of ALK-positive histiocytosis with *DCTN1::ALK* gene rearrangement. To date, only three such cases have been reported, with the present one being the first pediatric case [10,11,12].

Histiocytosis represents a histologically and clinically heterogenous group of conditions; according to Emile et al., the numerous entities can be classified into five major groups [13]. It is worth mentioning that this classification does not include ALK-positive histiocytosis as a separate entity among histiocytosis entities. The disease would fall into its subcategory, as it is not the disease of Langerhans cells (“L” Group), does not meet the criteria of either Rosai–Dorfman disease (“R” Group), or hemophagocytic lymphohistiocytosis (“H” Group), is devoid of anaplastic histology (“M” group), and does not strictly manifest in a cutaneous fashion (“C” Group). It is worth noting that there can be a significant overlap between the subgroups and entities. In our case, differential diagnoses included Spitz nevus and other members of the non-LCH subgroups, e.g., juvenile and adult xanthogranuloma, necrobiotic xanthogranuloma, benign cephalic histiocytosis, progressive nodular histiocytosis, and cutaneous Rosai–Dorfman disease. Our case lacked the histological hallmark of the xanthogranulomatous lesions; it was devoid of foamy macrophages; in addition, melanocytic markers (e.g., S-100, HMB-45) were negative in the lesional cells. CD163 immunopositivity proved histiocytic origin.

The different types of histiocytosis represent different age predilections: whereas Langerhans cell histiocytosis, benign cephalic histiocytosis, and juvenile xanthogranuloma are more prevalent in childhood, Erdheim–Chester disease and progressive nodular histiocytosis typically affect the elderly. In a recent literature review by Phillips et al., 26/35 cases of ALK-positive histiocytoses involved patients under the age of 18 years, 15 of whom were under 1 year [10]. With our patient being only 6 months old, our case represents the group of pediatric manifestations.

In all the cases that presented with skin involvement, the cutaneous manifestation of the disease was seen in various forms: solitary or diffuse papules, elevated nodules, and erythematous rash-like lesions. In our case, a solitary papule was seen on the left forearm. In other cases with mono-systemic skin manifestations, which were similar to our case, mainly solitary lesions were found, e.g., a nasal skin papule, a nodular scalp lesion, a single nodule on the right breast, or a papular back lesion [11,14,15]; however, one study reported diffuse, firm, red-brown papulosis [10], and in another two cases, diffuse mucocutaneous red papules were seen [16].

The *DCTN1* gene is located on chromosome 2 and encodes subunit 1 of dynactin, an essential cofactor of dynein-1, a cytoplasmic microtubule motor protein [17]. To date, over 30 point mutations in the gene have been associated with the spectrum of DCTN1-related neurodegeneration, which exhibits a phenotypic spectrum, including Perry syndrome, frontotemporal dementia, amyotrophic lateral sclerosis, and progressive supranuclear palsy [18].

Translocations and gene fusions involving *DCTN1* are, however, associated with other types of diseases. The most common and clinically significant fusion partner is *ALK*, which is also located on chromosome 2. *DCTN1::ALK* gene fusion was reported in a series of lung adenocarcinomas, sclerosing rhabdomyosarcoma, inflammatory myofibroblastic tumor, myelomonocytic leukemia, pancreatic ductal adenocarcinoma, glioblastoma, epithelioid fibrous histiocytoma, atypical Spitz tumor, and three cases of ALK-positive histiocytoses [10,11,19,20,21,22,23,24,25,26,27]. The breakpoint in the *DCTN1* gene usually involves exon 26. However, exon 20 and 16 breakpoints have also been reported in the literature [11,20,28,29]. In the case of *ALK* gene breakpoints, exon 20 is the most affected region, leaving the tyrosine kinase domain (exon 20–28) intact, allowing constitutive kinase activity-driven oncogenesis [30].

All three of the reported ALK-positive histiocytoses with *DCTN1::ALK* rearrangements were adult cases: a 32-year-old male, a 41-year-old female, and a 56-year-old male. The two male patients had widespread disease manifestation: liver, gallbladder, skin, lung, kidney, lymph node involvement, and widespread skin, bone, and soft tissue lesions. The female patient had a solitary right clavicular lesion with extension to surrounding soft tissue. To the best of our knowledge, our case is the first pediatric case and the first case with cutaneous-only involvement, harboring this specific gene fusion. Along with our case, in two cases the *DCTN1* breakpoint was found in exon 26; in the remaining case, no breakpoint was mentioned in the article. Due to the systemic involvement or locally advanced disease, all three patients were put on systemic kinase inhibitors, notably alectinib, with regressive disease upon 2-year follow-up.

In summary, we present a rare case of ALK-positive histiocytosis with unusual *DCTN1* fusion. It seems that *DCTN1*, as a fusion partner of *ALK*, does not predispose one to a specific clinical manifestation, disease course, or histological appearance; however, due to the rarity of this tumor entity, it is crucial to perform a larger study to elucidate this issue. Thus, we recommend professionals to perform FISH and—if available—NGS with *ALK* gene coverage on histiocytosis samples, where ALK immunopositivity is present.

## Figures and Tables

**Table 1:** Details of immunohistochemical staining. CC1—cell conditioning 1; DAB—diaminobenzidine; HIER BERS2—heat-induced epitope retrieval bond epitope retrieval solution 2; IHC—immunohistochemistry; RT—room temperature; RTU—ready to use.

ANTIBODY						ANTIGEN RETRIEVAL		IHC STAINERS	
Name	Vendor	Host	Clone	Dil.	Incubation Time/Temp	Method	Time/Temp	Visualization System	Platform
**ALK (D5F300A0045) XP^®^**	Cell Signaling Technology Europe, B.V., Leiden, The Netherlands	rabbit	D5F3	1/200	16′/36 °C	CC1 solution, pH 8.5	48′, 100 °C	OptiView DAB Detection, 6396500001	VENTANA BenchMark ULTRA
**Phospho-p44/42 MAPK (Erk1/2) (Thr202/Tyr204)**			D13.14.4E		32′/36 °C		36′, 100 °C	Ultraview Universal DAB Detection Kit, 5269806001	
**Cyclin D1**	Roche (Hungary) Ltd., Budapest, Hungary		SP4-R	RTU			48′, 100 °C	OptiView DAB Detection 6396500001	
**CD163**	Sigma-Aldrich Chemie GmbH, Taufkirchen, Germany	mouse	MRQ-26	1/200			36′, 100 °C		
**CD34**	Leica Biosystems Nussloch GmbH, Nussloch, Germany		QBEnd/10	1/2000				Ultraview Universal DAB Detection Kit, 5269806001	
**S-100**			polyclonal	1/200	32′/RT		20′, 100 °C		
**CD10**			56C6	1/100	30′/RT	HIER BERS2, pH 9		Bond Polymer Refine Detection	Leica-Bond Max
**Ki-67**	Dako, an Agilent Technologies Company, Glostrup, Denmark		MIB1	1/200					

## Data Availability

The original contributions presented in this study are included in the article. Further inquiries can be directed to the corresponding author.

## Abbreviations

The following abbreviations are used in this manuscript:

ALK	Anaplastic lymphoma kinase
CSF1R	Colony-stimulating factor 1 receptor
CT	Computed tomography
ERK	Extracellular signal-regulated kinase
FISH	Fluorescence in situ hybridization
HMB-45	Human melanoma black
JAK	Janus kinase
LCH	Langerhans cell histiocytosis
MAPK	Mitogen-activated protein kinase
MBC	Molecular barcode
MRI	Magnetic resonance imaging
MTOR	Mammalian target of rapamycin
NGS	Next-generation sequencing
NPM	Nucleophosmin
NTRK1	Neurotrophic receptor tyrosine kinase 1
PI3K	Phosphatidylinositol 3-kinase
STAT	Signal transducer and activator of transcription
VAF	Variant allele frequency
